# STAT3 Pathway in Gastric Cancer: Signaling, Therapeutic Targeting and Future Prospects

**DOI:** 10.3390/biology9060126

**Published:** 2020-06-12

**Authors:** Milad Ashrafizadeh, Ali Zarrabi, Sima Orouei, Vahideh Zarrin, Ebrahim Rahmani Moghadam, Amirhossein Zabolian, Shima Mohammadi, Kiavash Hushmandi, Yashar Gharehaghajlou, Pooyan Makvandi, Masoud Najafi, Reza Mohammadinejad

**Affiliations:** 1 Department of Basic Science, Faculty of Veterinary Medicine, University of Tabriz, Tabriz 5166616471, Iran; dvm.milad73@yahoo.com; 2 Sabanci University Nanotechnology Research and Application Center (SUNUM), 34956 Tuzla, Istanbul, Turkey; alizarrabi@sabanciuniv.edu; 3 Center of Excellence for Functional Surfaces and Interfaces (EFSUN), Faculty of Engineering and Natural Sciences, Sabanci University, 34956 Tuzla, Istanbul, Turkey; 4 Department of Genetic Science, Tehran Medical Science Branch, Islamic Azad University, Tehran 1916893813, Iran; Sima.orouei@gmail.com; 5 Laboratory for Stem Cell Research, Shiraz University of Medical Sciences, Shiraz 7134814336, Iran; zarrin.vahideh2075@gmail.com; 6 Department of Anatomical sciences, School of Medicine, Student Research Committee, Shiraz University of Medical Sciences, Shiraz 7134814336, Iran; Ebrahimrahmani1374@gmail.com; 7 Young Researchers and Elite Club, Tehran Medical Sciences, Islamic Azad University, Tehran 1916893813, Iran; Fzr2000_0007@yahoo.com; 8 General Practitioner, Kerman University of Medical Sciences, Kerman 7616913555, Iran; Shima.mohamadi92@yahoo.com; 9 Department of Food Hygiene and Quality Control, Division of Epidemiology & Zoonoses, Faculty of Veterinary Medicine, University of Tehran, Tehran 1417414418, Iran; houshmandi.kia7@ut.ac.ir; 10 Department of Clinical Sciences, Faculty of Veterinary Medicine, University of Tabriz, Tabriz 5166616471, Iran; yasharqarehaghaj@gmail.com; 11 Institute for Polymers, Composites and Biomaterials (IPCB), National Research Council (CNR), 80125 Naples, Italy; pooyanmakvandi@gmail.com; 12 Radiology and Nuclear Medicine Department, School of Paramedical Sciences, Kermanshah University of Medical Sciences, Kermanshah 6715847141, Iran; 13 Pharmaceutics Research Center, Institute of Neuropharmacology, Kerman University of Medical Sciences, Kerman 7616911319, Iran

**Keywords:** signal transducer and activator of transcription 3 (STAT3), gastric cancer, microRNA, long non-coding RNA, cancer therapy, tumor microenvironment

## Abstract

Molecular signaling pathways play a significant role in the regulation of biological mechanisms, and their abnormal expression can provide the conditions for cancer development. The signal transducer and activator of transcription 3 (STAT3) is a key member of the STAT proteins and its oncogene role in cancer has been shown. STAT3 is able to promote the proliferation and invasion of cancer cells and induces chemoresistance. Different downstream targets of STAT3 have been identified in cancer and it has also been shown that microRNA (miR), long non-coding RNA (lncRNA) and other molecular pathways are able to function as upstream mediators of STAT3 in cancer. In the present review, we focus on the role and regulation of STAT3 in gastric cancer (GC). miRs and lncRNAs are considered as potential upstream mediators of STAT3 and they are able to affect STAT3 expression in exerting their oncogene or onco-suppressor role in GC cells. Anti-tumor compounds suppress the STAT3 signaling pathway to restrict the proliferation and malignant behavior of GC cells. Other molecular pathways, such as sirtuin, stathmin and so on, can act as upstream mediators of STAT3 in GC. Notably, the components of the tumor microenvironment that are capable of targeting STAT3 in GC, such as fibroblasts and macrophages, are discussed in this review. Finally, we demonstrate that STAT3 can target oncogene factors to enhance the proliferation and metastasis of GC cells.

## 1. Introduction

Gastric cancer (GC) is one of the leading causes of death worldwide and its incidence rate is different among nations [1,2,3]. It has been reported that Eastern Asia claims the highest incidence rate of GC and after that, Central/Eastern Europe has the second place. It seems that non-cardiac GC is prevalent in the aforementioned places and cardiac GC has a high incidence rate in North America, Australia and the United Kingdom [4]. It is worth mentioning that infections can lead to the emergence of GC. The most well-known example of an infection resulting in GC is *Helicobacter pylori* (*H. pylori*) infection. There is information about the involvement of Epstein–Barr virus (EBV) infection in GC development [5]. The alterations in lifestyle play a significant role in GC emergence. Among them, cigarette smoking, obesity, high salt intake and low vegetable consumption are of importance [5,6]. The metastatic nature of GC cells has made them a leading cause of death. In a recent study, it was revealed that patients with GC exhibit metastasis into neighboring and distant tissues, such as the liver, peritoneum, lung and bone [7]. So, it seems that GC is a multifactorial disorder and dealing with this life-threatening disorder requires an understanding of the factors involved in its development and malignancy.

To date, a large body of evidence has been provided about the factors leading to GC progression. It seems that complex molecular pathways are the most important ones in GC malignancy [8]. The identification of these pathways can pave the way to effective GC therapy. Thanks to experiments performed in this field, it has been reported that a high number of molecular pathways and their upstream and downstream mediators, can contribute to GC development [9,10,11]. Due to limitations in space, it is impossible to describe all these underlying pathways, but we briefly discuss them to shed some light on the complexity of these signaling networks. The long non-coding RNAs (lncRNAs) are considered as the most common upstream mediators of molecular signaling pathways, since they are capable of the regulation of different pathways that affect biological mechanisms such as apoptosis, proliferation differentiation and so on [12,13]. In GC cells, lncRNA LINC00511 reduces the expression of microRNA (miR)-124-3p to induce PDK4. This axis leads to an increase in the proliferation and progression of GC cells [14]. Notably, there are interactions between lncRNAs in GC. The lncRNA PTCSC3 is an onco-suppressor factor and its expression undergoes downregulation in GC cells. There is negative feedback between PTCSC3 and HOXA11-AS, so that the upregulation of PTCSC3 provides the conditions for the downregulation of HOXA11-AS, resulting in decreased invasion and proliferation of GC cells [15]. As mentioned earlier, GC cells have a high metastatic ability that is correlated with the poor prognosis of patients. The epithelial-to-mesenchymal transition (EMT) is a signaling pathway capable of enhancing the migration of cancer cells [16,17]. The non-coding RNAs are able to act as upstream mediators of EMT in cancer cells [18]. In GC cells, the Wnt/β-catenin signaling pathway induces EMT to ensure the migration and invasion of cancer cells. The miR-330-3p acts as an onco-suppressor factor, inhibiting EMT through Wnt downregulation, leading to a decrease in the metastasis of GC cells, and an improvement in prognosis [19]. It is suggested that the tumor microenvironment plays a significant role in the progression and immune evasion of GC cells. The secretion of inflammatory cytokines, such as IL-4 and IFN-γ, results in PD-L2 expression that in turn provides immune suppression, leading to the proliferation and survival of cancer cells [20]. The growth of GC cells relies on the activation of molecular pathways that promote glucose metabolism. Due to the uncontrolled proliferation of GC cells, these networks should be in precise regulation. The PI3K/Akt signaling pathway participates in the proliferation of cancer cells and its disruption can pave the way to growth suppression [21]. In GC cells, HBXIP induces the PI3K/Akt pathway to enhance glucose metabolism. The inhibition of the HBXIP/PI3K/Akt axis is correlated with an inhibition in the growth of GC cells [22]. Overall, the studies are in line with the fact that a complicated network of molecular pathways is involved in GC malignancy [23,24]. In addition to the identification of molecular pathways, there have been attempts to target them using anti-tumor compounds. It seems that anti-tumor compounds, such as chrysin, psoralen and tivatinib, are able to induce apoptosis in GC cells and suppress their angiogenesis and migration through the downregulation of vascular endothelial growth factor (VEGF) [25,26,27]. In the current review, we present a comprehensive discussion of the STAT3 signaling pathway in GC malignancy and its regulation by upstream mediators. Furthermore, we represent how anti-tumor drugs can target STAT3 in suppressing GC progression and metastasis.

## 2. STAT3 Signaling Pathway and Its Role in Pathological Events

Signal transducer and activator of transcription (STAT) proteins are mediators for transferring signals from the extracellular matrix into the nucleus [28,29]. Among transcription factors, the role of STAT proteins has been extensively examined [29,30,31,32]. There are seven members of STAT proteins and STAT3 is of importance due to its role in affecting genes involved in cancer progression and malignancy [33]. As a protein with 770 amino acids, STAT3 has six characteristic domains with special functions. The protein–protein interactions are mediated via the N-terminal domain of the STAT3 protein. The coil–coil domain induces a nuclear localization signal. The interaction of STAT3 with target genes is performed through a DNA-binding domain. Src homology-2 (SH2) participates in STAT3 dimerization by the identification of phospho-tyrosine in the target protein. The phosphorylation of the C-terminal transcription activation domain (TAD) at serine sites provides the assembly of STAT3 with other transcriptional activators [34,35,36]. This shows that the STAT3 structure has been designed for interacting with different molecules, and this has resulted in pleiotropic impacts on STAT3 signaling pathway.

**Figure 1:**
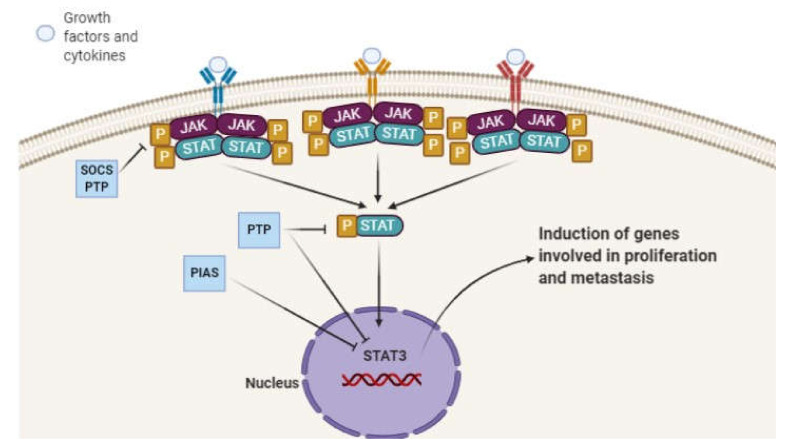
The schematic representation of the STAT3 signaling pathway.

The STAT3 signaling pathway is induced by the binding of an extracellular ligand, such as IL-6. This leads to the receptor dimerization and trans-phosphorylation/activation of Janus kinases (JAKs). The JAK proteins are involved in providing docking sites for STAT3 by the phosphorylation of cytoplasmic receptor tails. Then, JAK proteins activate the STAT3 signaling pathway via phosphorylation at tyrosine 705. After activation, STAT3 separates from the receptor/kinase complex to produce homodimers or heterodimers using its SH2 domain. Further signaling depends on translocation into the nucleus and targeting genes. Upon nuclear translocation, the STAT3 signaling pathway contributes to an enhancement in the proliferation, metastasis and migration of cancer cells. During physiological conditions, suppressor of cytokine signaling (SOCS) proteins, protein inhibitor of activated STAT (PIAS) proteins and phosphatases are able to negatively affect the expression of STAT3 and suppress its nuclear translocation. The SOCS3 interferes with JAK activity, while PIAS3 inhibits the STAT3–DNA interaction. The phosphatases, such as SHP-1, SHP-2 and PTP1B, can dually suppress JAK kinase activity or its nuclear interaction with target genes [37,38,39,40,41,42,43,44]. In cancer cells, the endogenous inhibitors of the STAT3 signaling pathway undergo downregulation, which mediates the enhanced proliferation and malignancy of cancer cells (Figure 1) [45,46,47,48].

Similar to other molecular signaling pathways, STAT3 can be considered as a downstream target. Proviral integration site for Moloney murine leukemia virus (PIM) kinases are a category of serine/threonine kinases and include PIM1, PIM2 and PIM3. PIM1 increases the proliferation and growth of cancer cells via the downregulation of cycle cell suppressor p27 [49]. In lung cancer cells, PIM1 stimulates STAT3 phosphorylation to suppress apoptosis [50]. The molecular pathways allowing the survival of cancer cells target STAT3. Stomatin-like protein 2 (SLP-2) is an oncogene factor that ensures the growth and viability of colorectal cancer cells via the upregulation of JAK2/STAT3 [51]. The lncRNAs are able to act as upstream mediators of STAT3 [52]. The lncRNA LINC01535 reduces the sensitivity of cancer cells to apoptosis and enhances their growth through the induction of the JAK/STAT3 signaling pathway [53]. The anti-tumor compounds can target the STAT3 signaling pathway in cancer therapy. For instance, *Centipeda minima* is a key member of Chinese traditional medicine (CTM) that suppresses the invasion and malignancy of breast cancer cells via the downregulation of the STAT3 pathway [54]. Brevilin A suppresses the phosphorylation of STAT3 at tyrosine 70 to restrict the growth of lung cancer cells [55]. These studies are in line with the fact that STAT3 is a tumorigenesis factor in cancer cells, and its inhibition is an ideal strategy in overcoming cancer (Table 1) [56,57,58,59].

## 3. Targeting STAT3 in Clinical Trials: A Focus on Cancer Therapy

In respect to the role of STAT3 in the development and progression of cancer, a variety of clinical trials have focused on targeting STAT3 in the treatment of patients with cancer. Table 2 summarizes the clinical trials related to targeting STAT3 in cancer therapy. In a study, AZD9150, as an inhibitor of STAT3, was administered for the treatment of patients with lymphoma and lung cancers. Before the introduction of AZD9150 in clinical trials, its efficacy was investigated in preclinical models of lymphoma and lung cancer. It effectively inhibited the proliferation and invasion of cancer cells. Then, it was translated into clinical trials. Similarly, in patients with lymphoma and lung cancer, AZD9150 demonstrated high anti-tumor activity [76], demonstrating its potential for being used in clinical trials. Notably, clinical trials have focused on using an expression profile of STAT3 as a prognostic factor. This study demonstrated that the expression of phosphorylated STAT3 (p-STAT3) is associated with the desirable prognosis of patients with luminal breast cancer. This phase III study puts an emphasis on the positive relationship between STAT3 and favorable prognosis [77]. Although this study provides controversial results that are not in line with our aforementioned statements, another study (104 French patients) also confirms that the expression of p-STAT3 is associated with the improved overall survival of patients with rectal cancer [78]. More studies are needed to clarify these discussions.

One of the challenges faced in radiotherapy is the resistance of cancer cells. Clinical trials have examined the relationship between STAT3 and the response of cancer cells to radiotherapy. It seems that STAT3 accumulation in cancer cells and its nuclear translocation can lead to radio resistance. In patients who are resistant into radiotherapy, genes associated with retinoid IFN-induced mortality-19 (GRIM-19) can inhibit STAT3 accumulation, resulting in enhanced sensitivity to radiotherapy [79].

Complicated molecular pathways are involved in ensuring the malignant behavior of cancer cells. In ovarian cancer, IL-6 induces STAT3 expression that in turn activates hypoxia inducible factor (HIF), resulting in the resistance of patients with ovarian cancer to chemotherapy with sunitinib [80]. Another clinical trial considered STAT3 as a factor involved in the migration of prostate cancer cells. In this study, 456 people were enrolled and it was found that STAT3 induction has a reverse correlation with distant metastasis, but it can lead to the local progression of prostate cancer cells [81].

## 4. Search Strategy

We conducted a literature search in different databases, such as PubMed, Scopus and Google scholar. Articles in English that were published before April 2020 were collected. The keywords included “STAT3 & gastric cancer”, “drug & STAT3 & gastric cancer”, “lncRNA & STAT3 & gastric cancer” and “miR & gastric cancer & STAT3”.

## 5. STAT3 and Gastric Cancer

### 5.1. MicroRNA-Mediated Regulation of STAT3

Although the STAT3 signaling pathway is an upstream mediator of a number of transcription factors, it can be regulated by miRs. Briefly, miRs are endogenous non-coding RNA with a low length of 19–23 nucleotides and can affect various biological processes via targeting different molecular pathways [84,85,86,87,88,89]. A high number of studies have demonstrated that miRs are able to regulate the STAT3 signaling pathway by targeting upstream mediators, such as ROCK1 [59], suppressing translation and phosphorylation [90], affecting JAK proteins [91] and influencing the nuclear translocation of STAT3 [92]. A similar phenomenon occurs in GC cells. It seems that miRs are able to target the STAT3 signaling pathway in GC to affect its metastasis and growth. In this way, miRs are divided into two categories, including onco-suppressor miRs, which reduce the expression of STAT3, while oncogene miRs are capable of enhancing the expression of STAT3. The miR-143 is considered as an onco-suppressor factor in GC, and its overexpression is associated with a decrease in survival and proliferation [93]. This miR can negatively affect both the proliferation and metastasis of GC cells via targeting molecular pathways such as DNMT3A and MYO6 [94,95]. In GC cells, miR-143 diminishes the expression of the STAT3 signaling pathway. Although expression of miR-143 undergoes downregulation in GC cells, enhancing the expression of this miR-143 paves the way for the downregulation of STAT3 and suppressing the invasion and proliferation of GC cells [96]. The miR-125a is another onco-suppressor miR whose downregulation mediates the undesirable prognosis of GC cells [97,98]. There is a reverse relationship between miR-125a and STAT3 in GC cells, as miR-125a reduces the expression of STAT3 to suppress the activation of its downstream target, HAS1, leading to a decrease in the migration and metastasis of GC cells [99]. As mentioned earlier, infection with *H. pylori* is one of the predisposing factors for GC development. This infection can lead to changes in immune responses and the enhanced production of inflammatory factors [100,101]. Accumulating data demonstrates that *H. pylori* infection results in the abnormal expression of miRs, which provide the conditions for GC development [102,103,104]. The miR-375 undergoes downregulation by *H. pylori* infection. Enhancing the expression of miR-375 is considered as a promising strategy in suppressing *H. pylori*-mediated GC development via the downregulation of the STAT3 signaling pathway. By the inhibition of STAT3, miR-375 suppresses the metastasis (Twist1 downregulation) and proliferation (Bcl-2 downregulation) of GC cells [105].

The miR-148a as an onco-suppressor factor that decreases the growth and invasion of GC cells and is capable of enhancing the sensitivity of GC cells to chemotherapy [106,107]. By the downregulation of the STAT3 signaling pathway, miR-148a suppresses cholecystokinin B receptor (CCK-BR) to interfere with the proliferation and migration of GC cells [108]. In respect to the high metastatic capability of GC cells, revealing the molecular pathways involved in their migration is of interest. Angiogenesis is a molecular mechanism that demonstrates overexpression in cancer cells [109,110]. In fact, based on the high growth and proliferation of cancer cells, they need a high amount of energy and oxygen supplies. By the induction of angiogenesis, cancer cells can promote their ability to proliferate. Besides, angiogenesis can enhance the migration of cancer cells [111,112,113]. Various molecular pathways have been considered as upstream mediators of angiogenesis, and STAT3 is one of them [114]. By the inhibition of STAT3, the induction of angiogenesis is inhibited and the growth of cancer cells is inhibited [115,116]. In GC cells, the STAT3 signaling pathway induces angiogenesis via the upregulation of VEGF. The miR-874 as an onco-suppressor factor that diminishes the expression of STAT3 to disrupt the STAT3/VEGF axis, leading to a decrease in the proliferation and migration of GC cells by the inhibition of angiogenesis [117]. In addition to angiogenesis, the STAT3 signaling pathway can regulate EMT in cancer cells. Increasing evidence demonstrates that the STAT3 pathway is capable of the induction of EMT in cancer cells, and in this way, a number of upstream mediators, such as miR-449b-3p and SIX4, act as inducers of STAT3/EMT [118,119]. The anti-tumor compounds inhibit EMT via STAT3 downregulation [120]. These studies exhibit the critical role of STAT3 in the regulation of EMT in cancer cells. The miR-216a has demonstrated great potential in suppressing the invasion and migration of GC cells via targeting the STAT3 signaling pathway. It seems that miR-216a inhibits the JAK2/STAT3 axis to downregulate EMT, resulting in a decrease in the metastasis of GC cells [121].

**Figure 2:**
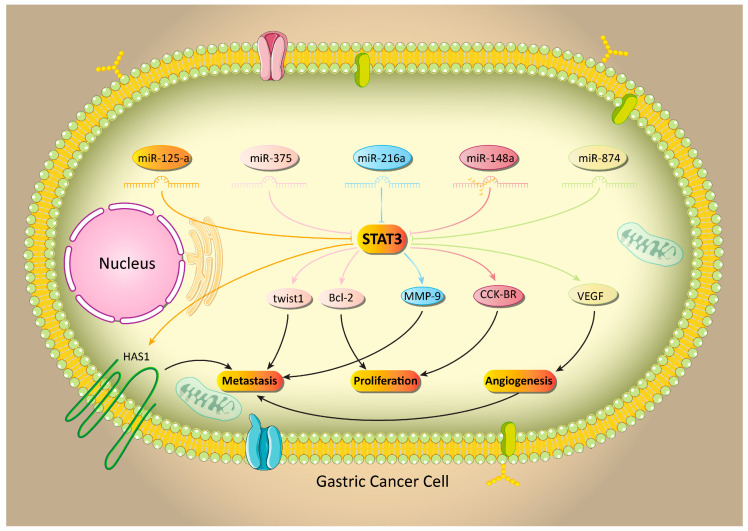
Onco-suppressor microRNAs in the inhibition of STAT3 and the malignant behavior of gastric cancer cells.

**Figure 3:**
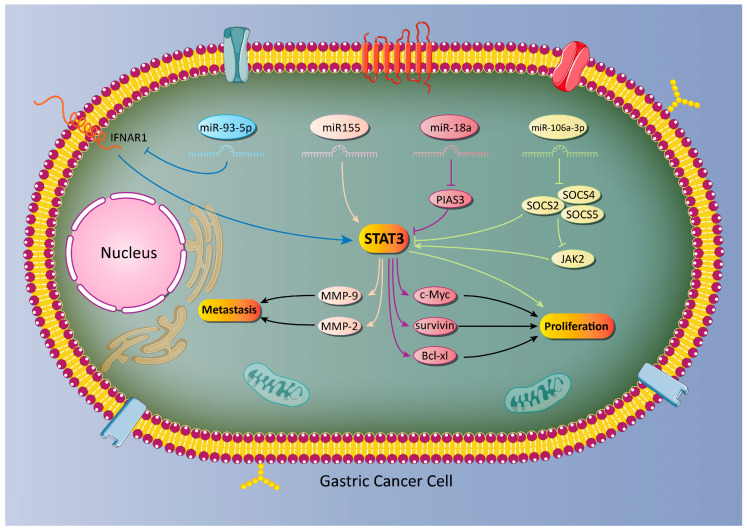
Oncogene miRs that activate the STAT3 signaling pathway and promote the proliferation and invasion of GC cells.

In addition to onco-suppressor miRs, the role of oncogene miRs in the regulation of STAT3 in GC has been examined. As mentioned earlier, EMT can enhance the migration of GC cells via transforming epithelial cells into mobile mesenchymal ones [122,123]. The matrix metalloproteinase proteins (MMPs) are also capable of promoting the metastasis of cancer cells via the degradation of the extracellular matrix (ECM) [124]. MMP-9 is a key member of this family and its role in GC has been investigated. It seems that MMP-9 upregulation enhances the metastasis of GC cells and is associated with an unfavorable prognosis [125,126]. The STAT3 signaling pathway induces MMP-9 to elevate the progression and metastasis of GC cells. The oncogene miR-93-5p inhibits STAT3 via IFNAR1 downregulation, leading to a decrease in MMP-9 expression and the malignant behavior of GC cells [127]. Notably, miRs are able to target endogenous inhibitors of the STAT3 signaling pathway, such as PIAS3, in cancer cells [128,129]. The miR-18a is suggested to be an oncogene factor in cancer and its downregulation can pave the way to suppressing cancer malignancy [113,130]. In GC cells, miR-18a down-regulates PIAS3 to induce the STAT3 signaling pathway. As a consequence, downstream targets of STAT3 including, c-Myc, Survivin and Bcl-xl, undergo upregulation that ensures the viability and proliferation of cancer cells [131]. Overall, studies are in agreement with the fact that miRs are potential upstream mediators of the STAT3 signaling pathway in GC and the modulation of the miR/STAT3 axis can lead to effective GC therapy (Table 3, Figure 2 and Figure 3).

### 5.2. Drug-Mediated Regulation of STAT3

Based on the role of the STAT3 signaling pathway in enhancing the progression and malignancy of GC cells, much attention has been directed towards the regulation and targeting of this pathway in GC therapy. Anti-tumor compounds are able to target STAT3 in GC therapy [134,135]. It is worth mentioning that, to date, most of the anti-tumor drugs applied in the treatment of GC cells by targeting STAT3 have been isolated from plants. The plant-derived natural compounds have demonstrated great potential in the regulation of the STAT3 signaling pathway in cancer therapy [136]. In GC therapy, the natural products capable of targeting the STAT3 signaling pathway have been applied. In Table 4, we summarize these anti-tumor compounds, and this section, we describe their ability in suppressing GC malignancy and proliferation. Cucurbitacins are anti-tumor compounds and well known in traditional Chinese medicine [137]. These plant-derived natural compounds have anti-tumor activity against GC cells and are able to suppress the growth and viability of GC cells via the induction of cell cycle arrest [138]. A newly published article has shed some light on the anti-tumor activity of cucurbitacins in GC cells. It seems that the STAT3 signaling pathway ensures the growth and survival of GC cells via the induction of Bcl-xl and c-Myc upregulation. The administration of cucurbitacin B is correlated with the downregulation of STAT3 and its downstream targets. In the inhibition of STAT3 activity, cucurbitacin B attaches to the DNA-binding domain of STAT3. This decrease in STAT3 activity and expression caused by cucurbitacin B paves the way for a reduction in the proliferation of GC cells and their sensitization into the anti-tumor activity of cisplatin as a chemotherapeutic agent [126]. This study highlights the fact that natural products can be used as chemosensitizers in GC therapy. The examination of molecular pathways demonstrates that anti-tumor compounds are able to enhance the generation of reactive oxygen species (ROS) to modulate the expression of STAT3. In fact, phytochemicals induce ROS production to stimulate apoptotic cell death via the downregulation of STAT3 phosphorylation [139]. This strategy is not only beneficial in the induction of apoptosis in GC cells, but it can also mediate the stimulation of cell cycle arrest. After the administration of glycitein as an anti-tumor agent, GC cells undergo cell cycle arrest at the G0/G1 phase. This anti-tumor activity partially emanates from the inhibitory effect of glycitein on the expression of STAT3 [140]. However, natural products with anti-tumor activity can reduce the viability of GC cells via the reduction of ROS levels. In fact, they are not just dependent on the induction of ROS generation. This is due to the dual role of ROS in cancer cells. Although ROS can mediate the mitochondrial dysfunction and stimulation of endoplasmic reticulum (ER) stress [141,142,143], accumulating data demonstrates that ROS can lead to tumorigenesis via the activation of oncogene signaling pathways, such as STAT3 and Wnt/β-catenin [144,145]. In these cases, decreasing ROS production can pave the way to effective cancer therapy. A similar strategy is used by lycopene as a potential anti-tumor agent [146]. In suppressing GC development, lycopene reduces ROS generation that in turn inhibits the STAT3 signaling pathway, as a carcinogenesis factor [147].

In cancer cells, IL-6 functions as an upstream mediator of STAT3 signaling pathway. It has been demonstrated that IL-6 can induce STAT3 to ensure the malignant behavior of cancer cells [148]. Apigetrin (APG) is a flavonoid compound with excellent anti-tumor activity [149]. In GC cells, the administration of APG induces apoptosis and remarkably diminishes their growth and proliferation. The examination of molecular pathways demonstrates that APG inhibits STAT3 through IL-6 downregulation. Besides, APG triggers the dephosphorylation of JAK2/STAT3 [150], making it a suitable compound in GC therapy. NF-kB is an oncogene signaling pathway responsible for the enhanced proliferation and migration of cancer cells [151]. Increasing evidence demonstrates that there is a dual relationship between NF-kB and STAT3 through the p65 and p50 subunits [152,153]. Targeting the STAT3/NF-kB axis is a potential strategy in cancer therapy [154]. Troxerutin (TXN) is a natural flavonoid rutin with different pharmacological activities, such as anti-diabetes, hepatoprotective, neuroprotective, antioxidant and anti-inflammatory [155,156]. Newly recorded studies are in line with the fact that TXN is capable of affecting molecular pathways, such as NF-kB and MDM2, in cancer therapy [157,158]. TXN supplementation is associated with a decrease in the survival of GC cells and their sensitivity to 5-fluorouracil chemotherapy. It is said that TXN is able to suppress STAT3 phosphorylation, which subsequently reduces the expression of NF-KB, leading to the decreased viability and growth of GC cells [159].

In previous section, we mentioned that miRs can function as upstream regulators of the STAT3 signaling pathway in cancer cells. The accumulated data demonstrate that the STAT3 signaling pathway can also regulate miR expression as an upstream mediator [160,161]. This crosstalk is of importance in cancer cells. The miR-373 is an oncogene factor capable of increasing the malignancy and proliferation of cancer cells via targeting molecular pathways. This miR dually promotes metastasis and proliferation, and its inhibition can remarkably suppress cancer malignancy, leading to their sensitivity to chemotherapy [162,163,164]. Exposing GC cells to isoproterenol sensitizes GC cells to cell death. Isoproterenol inhibits STAT3 phosphorylation to suppress miR-373 expression, resulting in the inhibition of drug resistance and metastasis via the upregulation of E-cadherin [165]. As discussed earlier, *H. pylori* can cause a predisposition to GC. It has been reported that *H. pylori* stimulates the STAT3 signaling pathway in GC development. The administration of docosahexaenoic acid (DHA) induces peroxisome proliferator-activated receptor gamma (PPAR-γ) to inhibit the phosphorylation of STAT3 at tyrosine 705. Besides, DHA enhances the expression of SOCS3 and suppresses the nuclear translocation of STAT3, resulting in a decrease in the proliferation and invasion of GC cells [166]. Taking everything into account, studies are in agreement with the fact that anti-tumor compounds are able to inhibit STAT3 in different stages, including targeting upstream mediators, the activation of endogenous inhibitors, the downregulation of downstream targets and suppressing STAT3 expression [167,168,169,170,171,172,173,174].

### 5.3. LncRNA-Mediated Regulation of STAT3

**Figure 4:**
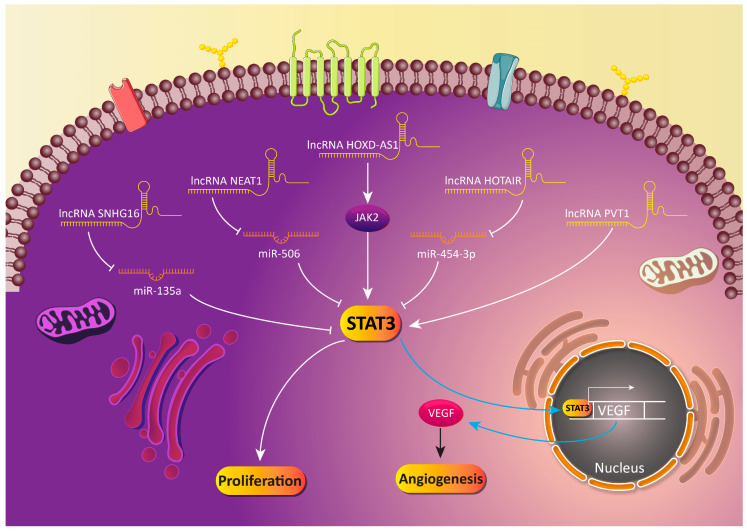
Long non-coding RNAs and their downstream targets in the regulation of the STAT3 signaling pathway in GC cells.

The lncRNAs are key members of non-coding RNAs with lengths more than 200 nucleotides [196]. Similar to miRs, lncRNAs are able to affect and regulate a number of biological mechanisms, such as cell proliferation, differentiation, angiogenesis, migration and so on [197,198]. These modulatory effects of lncRNAs have led to the investigation of their roles in different diseases, particularly cancer [199,200]. Newly published studies have shown that lncRNAs are able to target molecular pathways by the induction of their effects [201,202]. It is worth mentioning that a large body of evidence has examined the relationship between lncRNAs and the STAT3 signaling pathway [203,204]. The oncogene lncRNAs are able to upregulate the expression of STAT3, while onco-suppressor lncRNAs reduce the expression of STAT3 [205,206,207,208]. It is held that targeting the lncRNA/STAT3 axis is of importance in cancer therapy [209,210]. Fortunately, experiments have attempted to provide information about the dual relationship between lncRNAs and STAT3 in GC cells, and it has been shown that not only can lncRNAs function as upstream regulators of STAT3, but also STAT3 can affect the expression of lncRNAs [211,212]. The identification of this feedback is of importance in effective GC therapy. To date, just oncogene lncRNAs targeting STAT3 and their regulation have been investigated in GC. The lncRNA SNHG16 is an oncogene factor that is correlated with the invasion and growth of cancer cells. This lncRNA enhances the migration of cancer cells via the induction of EMT. Besides, the lncRNA SNHG16 regulates miRs in exerting their stimulatory effect on cancer cells [213,214,215]. In GC cells, SNHG16 considerably reduces the expression of the onco-suppressor miR-135a to activate the JAK2/STAT3 signaling pathway. This leads to an increase in colony formation and the growth of GC cells and reduces their sensitivity to apoptotic cell death [195]. Based on the role of oncogene lncRNAs in promoting the malignant behavior of GC cells, their modulation can pave the way into effective cancer therapy. It is held that the downregulation of the lncRNA HOTAIR, as an oncogene factor, enhances the expression of miR-454-3p. This miR is able to negatively affect the survival of GC cells by the induction of apoptosis and cell cycle arrest partially via the inhibition of the STAT3/cyclin D1 axis [216]. As mentioned earlier, STAT3 can induce angiogenesis via targeting VEGF. This results in an increase in the proliferation and viability of cancer cells [217,218]. It is held that lncRNAs can regulate the STAT3/VEGF axis in GC cells to affect angiogenesis and their proliferation. The lncRNA PVT1 is an oncogene lncRNA that induces angiogenesis via the activation of the STAT3/VEGF axis. This axis and the stimulation of angiogenesis are positive factors for the enhanced proliferation and migration of GC cells [219]. These studies demonstrate that lncRNAs indirectly affect the STAT3 signaling pathway by targeting their upstream mediators, such as miR-506 and IL-6. It is worth mentioning that the dual relationship between lncRNAs and the STAT3 signaling pathways can lead to the increased malignant behavior of GC cells, since they can increase their expression in a positive feedback loop [220,221]. Further studies should focus on revealing more oncogene lncRNAs, and also onco-suppressor lncRNAs. Besides, the genetic or pharmacological targeting of lncRNAs can pave the way to effective GC therapy (Table 5, Figure 4).

### 5.4. Other Molecular Signaling Pathways Regulate STAT3

In previous sections, we provided explanations about the role of upstream mediators, such as miRs and lncRNAs, in the regulation of the STAT3 signaling pathway in GC cells. Besides, we demonstrated that anti-tumor compounds are able to target the STAT3 signaling pathway in suppressing GC proliferation and malignancy. In this section, we discuss the other molecular pathways capable of targeting the STAT3 signaling pathway in GC cells. The identification of these signaling pathways and their upstream and downstream targets can pave the way to effective GC therapy. Sirtuins are a family of histone deacetylases and their function relies on NAD^+^. This family consists of seven families (SIRT1-7) and they are able to modulate different biological mechanisms, including cell metabolism, cell division and aging [224]. Increasing evidence demonstrates that SIRT1 is a positive factor for the progression and growth of GC cells. It has been reported that SIRT1 upregulation is correlated with reduced survival and undesirable prognosis [225]. However, another study exhibits that SIRT1 suppresses GC growth and stimulates apoptosis and cell cycle arrest in GC cells [226]. In GC cells, the STAT3 signaling pathway increases the invasion and migration of cancer cells via the stimulation of MMP-13. It is held that SIRT1 downregulates STAT3 to inhibit MMP-19 expression, resulting in the decreased invasion and malignancy of GC cells [227]. SIRT6 is another member of the SIRT family and is capable of reducing the proliferation of GC cells. The previous study revealed that SIRT1 exerts an inhibitory effect on the migration and metastasis of GC cells. It appears that SIRT6 negatively affects the proliferation and growth of GC cells by the inhibition of the STAT3 signaling pathway and the subsequent downregulation of cyclin D1 and Bcl-2 [228].

Stathmin (STMN) is a microtubule-regulating protein capable of the regulation of mitosis via targeting the aggregation and depolymerization of spindles. The STMN is an oncogene factor that undergoes upregulation in different tumors to ensure their proliferation and viability. Besides, STMN can be considered as a potential factor for the diagnosis of cancer [229,230,231]. The relationship between STMN and the STAT3 signaling pathway is of importance in GC cells. It has been reported that STMN dually enhances the migration and growth of GC cells. The knock-down of STMN reduces the expression of STAT3, resulting in cell cycle arrest and apoptosis in GC cells [232]. The uncontrolled proliferation of cancer cells requires a vast source of energy. Increasing evidence demonstrates that cancer cells enhance their glucose metabolism to meet their energy needs. This is known as the Warburg effect [233,234]. The examination of molecular pathways reveals interesting pathways involved in the Warburg effect of GC cells. PKM2 and c-Myc are considered as factors involved in the glycolysis of GC cells. These factors provide the mild acidic pH of the tumor microenvironment and promote glucose metabolism to provide for the proliferation of GC cells. STAT3 acts as an upstream mediator of c-Myc, while mTOR is the upstream mediator of PKM2. More importantly, it seems that the STAT3/c-Myc and mTOR/PKM2 signaling pathways have positive feedback and together they can lead to the enhanced proliferation and energy metabolism of GC cells [235]. The downregulation of the aforementioned signaling networks can result in the growth inhibition of GC cells.

RNF6 is a member of the E3 ligase family and its role in cancer has been explored. This oncogene factor is able to promote the growth and viability of cancer cells via the upregulation of pro-survival factors, such as Bcl-xl and Mcl-1 [236]. The clinical trials have also confirmed the role of RNF6 in the malignancy of cancer cells. It has been shown that RNF6 overexpression can mediate the metastasis and migration of colorectal cancer cells [237]. So, recognizing the downstream targets of RNF6 is of importance in cancer therapy. There is a dual relationship between RNF6 and the STAT3 signaling pathway in GC cells. RNF6 induces the STAT3 signaling pathway to upregulate the expression of pro-survival factors, such as XIAP and Mcl-1, resulting in an increase in the growth and survival of GC cells [238]. The signaling networks not only ensure the survival and proliferation of GC cells, but they can also trigger the resistance of GC cells to chemotherapy. Sphingosine-1-phosphate receptor (S1PR1) is a member of the G protein-coupled receptor family that is able to promote the malignant behavior of cancer cells. Onco-suppressor factors, such as miR-125b-1-3p, downregulate the expression of S1PR1 to suppress the migration and invasion of cancer cells and induce apoptosis [239]. It is held that the overexpression of S1PR1 is associated with chemoresistance [240]. The relationship between S1PR1 and STAT3 mediates the resistance of GC cells to chemotherapy. The inhibition of the S1PR1/STAT3 axis is correlated with the sensitization of GC cells to chemotherapy [241]. There is evidence that the inhibition of two signaling pathways is absolutely efficient in suppressing the chemoresistance of GC cells. Y-box binding protein-1 (YB-1) is capable of mediating the chemoresistance of cancer cells [242]. On the other hand, increasing evidence has demonstrated that the STAT3 signaling pathway is involved in chemoresistance [243]. In GC cells, the inhibition of STAT3 and YB-1 can suppress the resistance of cancer cells to chemotherapy. Although there is no dual relationship between STAT3 and YB-1, it has been reported that their simultaneous inhibition can result in synergistic effects in sensitizing GC cells to chemotherapy [244]. It is worth mentioning that, in addition to chemoresistance, the STAT3 signaling pathway may trigger the immune evasion of cancer cells. The PD-1/PD-L1 axis undergoes overexpression in malignant tumors and it can be regulated by different factors, such as miRs, lncRNAs, transcription factors and so on [245,246,247,248]. The enhanced expression of PD-1 provides the conditions for the resistance of cancer cells to chemotherapy and is correlated with undesirable prognosis [249,250,251]. It is said that GC mesenchymal stem cells are able to elevate levels of IL-8. IL-8 functions as an upstream mediator and upregulates the expression of STAT3. The STAT3 signaling pathway induces c-Myc to activate the PD-1/PD-L1 axis, leading to immunosuppression and the increased malignant behavior of GC cells [252].

TMEM119 is a member of the transmembrane proteins with important functions in cancer cells. TMEM119 provides the unfavorable prognosis of a patient with prostate cancer [253]. As a consequence, targeting TMEM119 is a potential strategy in overcoming cancer [254]. A same story occurs in GC cells. It is held that TMEM119 enhances the invasion and metastasis of GC cells via the upregulation of the STAT3 signaling pathway [255]. The inhibition of TMEM119 paves the way to GC treatment. It seems that STAT3 can trigger EMT in GC cells. However, upstream mediators are able to affect the STAT3/EMT axis. Nuclear factor I/B (NFIB) participates in normal somatic development and recent studies have revealed its role in cancer progression and development [256,257]. NFIB is able to induce Akt phosphorylation. Subsequently, an increase occurs in the expression of STAT3, resulting in the activation of EMT by enhancing vimentin levels and decreasing E-cadherin levels [258]. A same phenomenon occurs during inflammation. Increasing evidence shows that chronic inflammation can remarkably promote the progression and malignancy of cancer cells [259,260]. IL-23 is one of the pro-inflammatory cytokines and it has been reported that IL-23 induces the EMT mechanism through STAT3 upregulation to ensure the migration and metastasis of GC cells [261].

The tumor microenvironment (TME) is a complicated structure, consisting of different cells that have been included in the ECM [262]. Fibroblasts, endothelial cells, pericytes, immune cells and inflammatory cells are the main components of the TME. The bidirectional communication among these cells plays a significant role in cancer progression and malignancy [263]. Cancer-associated macrophages (CAMs) are M2 phenotype macrophages and an increase in their number is associated with poor prognosis in different cancers, particularly GC [264,265,266,267]. The CAMs are able to secrete IL-10 that in turn activates the c-Met signaling pathway. The c-Met acts as an upstream mediator of STAT3, resulting in the enhanced proliferation and invasion of GC cells [268]. Tumor-associated macrophages (TAMs) are one of the most important and abundant components of the TME. These macrophages have tumor-promoting effects (M2 phenotype macrophage) and are CD68-marked [269,270,271,272]. The TAMs enhance the levels of pro-inflammatory cytokines, including IL-6 and IL-8, to activate the STAT3 signaling pathway, leading to the metastasis and progression of GC cells [273]. Experiments also demonstrate that pro-inflammatory cytokines in the TME can provide the conditions for the differentiation of macrophages into the M2 phenotype that exerts a stimulatory effect on the proliferation and progression of cancer cells. IL-6 enhances levels of M2 macrophages with a high expression of IL-10 and TGF-β. It is held that the effect of IL-6 on macrophage differentiation is mediated through STAT3 upregulation. This axis leads to a remarkable increase in the progression and malignancy of GC cells [274]. More importantly, the TME can trigger the resistance of cancer cells to chemotherapy and immunotherapy. This has resulted in considerable attention to role of the TME in cancer malignancy [275,276,277]. Cancer-associated fibroblasts (CAFs) are another key member of the TME that can induce the metastasis, growth and malignancy of cancer cells [278]. In GC cells, CAFs secrete IL-11 that in turn activates the JAK/STAT3 signaling pathway. This leads to an increase in expression of anti-apoptotic factor Bcl-2 to mediate the resistance of GC cells to chemotherapy [279].

Taking everything into account, studies are in agreement with the fact that different signaling pathways can function as upstream mediators in the induction of STAT3 in GC cells. The identification of these pathways and further targeting can pave the way to effective GC therapy [280,281,282,283,284]. Besides, we demonstrated that different components of the TME are able to secrete inflammatory cytokines to activate STAT3, ensuring the metastasis and invasion of GC cells (Table 6).

## 6. STAT3 as an Oncogene Factor in Gastric Cancer

In previous sections, it was revealed that lncRNAs, miRs and other molecular pathways are able to regulate the STAT3 signaling pathway in GC cells. Besides, we described how anti-tumor compounds are able to modulate STAT3. In this section, we demonstrate that STAT3 can function as an upstream mediator to affect other molecular pathways, leading to the malignant behavior of GC cells [304,305]. DNA methylation and histone modification are hallmarks of carcinogenesis and they are able to influence the transcription output of the genome [306,307]. It is held that the presence of p-H3S10 and/or H3S28ph in the promoter regions can accelerate histone modification [308]. In GC cells, the STAT3 signaling pathway stimulates epigenetic kinase mitogen- and stress-activated protein kinase 1 (MSK1) to induce H3S10 phosphorylation, resulting in an increase in the tumorigenesis of GC cells [309]. A recently recorded study shows that the STAT3 signaling pathway can cause the poor differentiation of GC cells and is correlated with their metastasis into distant tissues [310]. Interestingly, downstream targets of the STAT3 signaling pathway can be considered as biomarkers for the early detection of GC. STAT3 is able to inhibit SPG20 expression via hypermethylation and the downregulation of this factor is a potential biomarker for GC detection [311]. It is worth mentioning that polymorphisms in STAT3 are correlated with GC tumorigenesis. It appears that the existence of a minor allele of STAT3 (rs1053023) is associated with a risk of GC development [312]. These studies demonstrate that not only downstreams of STAT3 can be used as biomarkers for GC detection, but also own STAT3 is a potential biomarker in GC identification. The clinical studies are also in line with the benefit of using STAT3 as a diagnostic factor. It has been reported that a positive feedback loop between STAT3 and miR-200 can promote the progression of GC cells, and this feedback is of importance for the diagnosis and prognosis of GC [313]. Overall, the overexpression of STAT3 provides the poor prognosis of patients with GC and its expression undergoes upregulation at the point when the gastric mucosa reaches the tumor stage [314,315]. In enhancing the growth and viability of GC cells, STAT3 targets cell cycle proteins. STAT3 is able to induce cyclin D1 expression, resulting in an increase in the proliferation and malignancy of GC cells [316].

CD163 is called a macrophage-associated antigen and is abundantly expressed in monocytes and macrophages [317]. CD163 has physiological functions, such as iron metabolism and the endocytosis of hemoglobin–haptoglobin complexes [318]. In spite of these vital functions in normal conditions, increasing evidence demonstrates that CD163 may be involved in the emergence of different disorders, particularly cancer [319,320,321]. STAT3 enhances the expression of CD163 on the components of the TME, such as macrophages, to enhance the proliferation of GC cells [322]. Targeting CD163 can be considered as a potential strategy in effective GC therapy. CD44 is another factor that can be targeted in GC cells by the STAT3 signaling pathway. CD44 is a cell adhesion molecule and this trans-membrane glycoprotein increases the proliferation and metastasis of cancer cells by binding to hyaluronic acid [323,324,325]. Accumulating data demonstrates that CD44 is a cell surface marker of cancer stem cells and is of importance for the malignancy of GC cells [324,325,326,327,328,329]. CD44 undergoes upregulation by STAT3 in GC cells and provides the undesirable prognosis of patients with GC [292].

**Figure 5:**
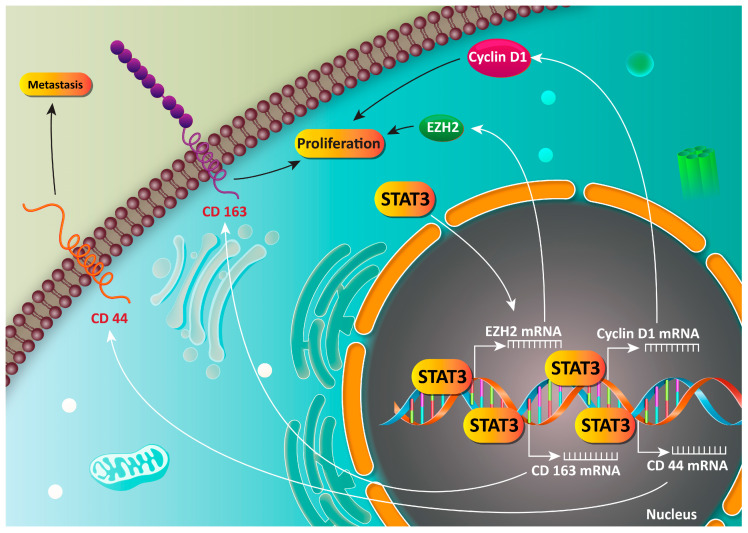
STAT3 signaling pathway as an oncogene factor in GC cells.

Enhancer of zeste homologue 2 (EZH2) is a key member of the polycomb group genes and is able to promote the malignancy of cancer cells via inhibiting the expression of a variety of tumor suppressor genes (TSGs) [330]. EZH2 dually enhances the migration and proliferation of cancer cells, and targeting this oncogene factor is a promising strategy in cancer therapy [331,332]. In GC cells, STAT3 functions as an upstream mediator of EZH2. By the activation of EZH2, STAT3 elevates the proliferation and metastasis of GC cells, and is correlated with poor prognosis [333]. In respect to the oncogene role of STAT3 in GC, studies have focused on investigating the expression of STAT3 in GC cells. It seems that the expression of STAT3 undergoes overexpression in gastric stromal tumors, and this can be considered as a diagnostic and prognostic factor (Figure 5) [334,335,336,337,338,339,340].

## 7. Conclusions and Remarks

The STAT3 signaling pathway is a well-known oncogene factor in different cancers, and its involvement in the malignancy and growth of GC cells has been extensively investigated. The present review focused on revealing the downstream and upstream mediators of STAT3 in GC cells to pave the way to understanding the oncogene pathways in this malignant tumor. Oncogene miRs and lncRNAs induce the STAT3 pathway, while onco-suppressor ones inhibit the STAT3 signaling pathway. We devoted a section to examining the relationship between anti-tumor compounds and the STAT3 signaling pathway in GC cells. It was revealed that anti-tumor agents are able to suppress STAT3, resulting in a decrease in the proliferation and invasion of GC cells. One of the interesting points about the STAT3 signaling pathway is that inflammatory factors can act as upstream mediators of STAT3. This relationship is of importance in the tumor microenvironment, since present cells are able to secrete interleukins that in turn activate the STAT3 signaling pathway, leading to the elevated progression and malignancy of GC cells. Finally, we demonstrated that STAT3 can be considered as a diagnostic and prognostic factor in GC. Although studies have extensively examined the molecular pathways involved in the STAT3 regulation in GC, and how anti-tumor compounds can be beneficial in suppressing STAT3 in GC therapy, there are a number of drawbacks that should be considered in further studies. Small interfering RNA (siRNA) has been applied in the inhibition of STAT3 and improving the prognosis of GC [341]. However, the off-targeting of siRNA, and also its degradation have limited its efficacy. On the other hand, anti-tumor compounds and their ability in GC therapy should be improved, since they have low bioavailability, and there are impediments to their entrance into cancer cells, such as the blood–tumor barrier. Recently, nanoparticles have gained significant attention in cancer therapy [342,343]. Using nanoparticles for the encapsulation of siRNA and anti-tumor compounds can facilitate the way to effective GC therapy, as the efficacy of siRNA-loaded nanoparticles improves gene silencing and the high cellular uptake of anti-tumor compounds is observed after nanoparticle delivery.

## Figures and Tables

**Table 1:** The role and regulation of STAT3 in different cancers.

Cancer Type	Signaling Network	Effect on STAT3	Results	Refs
Breast cancer	BHLH40-AS1/IL-6/STAT3	Induction	Promoting progression and proliferation	[60]
	IL-6/STAT3	Induction	Radiation induces STAT3-mediated inflammation and radio resistance	[61]
	MiR-454/VGLL4/STAT3	Induction	MiR-454 induces the STAT3 signaling pathway via VGLL4 downregulation, leading to cancer malignancy	[62]
	PAK1/STAT3	Induction	Stimulation of the nuclear translocation of STAT3 and enhancing breast cancer stem cell proliferation	[63]
	SIRT4/IL-6/STAT3	Inhibition	Sensitizing cancer cells to tamoxifen chemotherapy	[64]
Prostate cancer	IL-8/STAT3/MALAT1	Induction	STAT3 upregulates the expression of MALAT1, leading to progression and proliferation	[65]
	MiR-17/JAK/STAT3	Inhibition	MiR-17 reduces the expression of pro-survival factors, such as Bcl-2, and induces apoptosis via STAT3 downregulation	[66]
Bladder cancer	MiR-4500/STAT3/CCR7	Inhibition	Suppressing migration and proliferation	[67]
	CD44/Akt/ERK/STAT3	Induction	Inhibition of apoptosis and cell cycle arrest	[68]
Lung cancer	B7-H4/PD-1/STAT3	Induction	Promoting proliferation and invasion via immune evasion	[69]
	KCP10043F/STAT3	Inhibition	Induction of apoptotic cell death	[70]
	BIS/STAT3	Induction	Reducing sensitivity of cancer cells to digoxin-mediated migration and growth inhibition	[71]
Glioblastoma	Annexin-A2/STAT3/oncostatin M receptor	Induction	Promoting the proliferation and invasion of cancer cells	[72]
	Hsa-miR-181d/STAT3	Inhibition	Garcinol upregulates the expression of hsa-miR-181d to inhibit STAT3 and the malignancy of cancer cells	[73]
	TROP2/JAK2/STAT3	Induction	Promoting proliferation and migration	[74]
	Bradykinin B1 receptor/STAT3/IL-8	Induction	Enhancing malignant behavior	[75]

**Table 2:** Targeting STAT3 in cancer therapy in clinical trials.

Drug/Molecular Pathway	Effect on STAT3	Clinical Trial Phase	Major Outcomes	Refs
AZD9150	Inhibition	Phase I	Anti-tumor activity in pre-clinical models and clinical trial	[76]
GRIM19	Inhibition	-	Sensitizing into radiotherapy	[79]
-	-	Phase I	STAT3 provides local progression	[81]
Nilotinib	Inhibition	Phase II	Diminution in cancer growth	[82]
OPB-31121	Inhibition	Phase I	High tolerance Inhibition of tumor growth	[83]

**Table 3:** The regulation of STAT3 by miRs in gastric cancer.

MiiR	MiR Type	Cancer Cell Line	Effect on STAT3	Major Outcomes	Refs
MiR-143	Onco-suppressor	GC cell lines (AGS, SNU-1, SNU-5, SNU-16, NCIN87 and KATOIII)	Downregulation	Disrupting the proliferation and invasion of cancer cells	[96]
MiR-125a	Onco-suppressor	Human GC cell lines MKN45, SGC7901 and NCI-N87	Downregulation	Reducing the expression of HAS1 and interfering with the migration of cancer cells	[99]
MiR-375	Onco-suppressor	Human GC cell lines BGC-823, AGS, SGC-7901 and MKN-45	Downregulation	Inhibiting proliferation and migration via STAT3 downregulation	[105]
MiR-148a	Onco-suppressor	GC cell lines SNU-1 (ATCC: CRL-5971), SNU-16 (ATCC: CRL-5974), AGS (ATCC: CRL-1739), NCI-N87 (ATCC: CRL-5822) and KATOIII (ATCC: HTB-103)	Downregulation	Suppressing growth and metastasis by the downregulation of CCK-BR via STAT3 downregulation	[108]
MiR-874	Onco-suppressor	Human GC cell lines AGS and BGC823, MKN28 and SGC-7901, as well as the human normal gastric epithelial cell line GES-1	Downregulation	Disrupting the STAT3/VEGF axis Inhibition of angiogenesis and the malignancy of cancer cells	[117]
MiR-216a	Onco-suppressor	Normal human gastric epithelium cell line (GES-1) and GC cell lines (SGC-7901, MGC-803, MKN-28 and BGC-823)	Downregulation	Suppressing the metastasis of cancer cells via disrupting the STAT3/EMT axis	[121]
MiR-93-5p	Oncogene	AGS and HEK293 cells	Upregulation	Downregulation of the STAT3/MMP-9 axis Inhibition of metastasis and invasion	[127]
MiR-18a	Oncogene	Human GAC cell lines MKN28 and MKN1	Upregulation	STAT3 induction Promoting the malignant behavior of cancer cells	[131]
MiR-155	Oncogene	Human GC cell lines BGC-823, NCI-N87, SGC-7901, AGS, MKN-45 and immortalized gastric mucosa epithelial cell line GES-1	Upregulation	Stimulation of STAT3 Activation of MMP-2 and MMP-9 Enhancing invasion and migration of cancer cells	[132]
MiR-106a-3p	Oncogene	Human GC cell line including SGC-7901 and BGC-823	Upregulation	Stimulation of aptinib resistance Activation of JAK2/STAT3 signaling SOCS2, SOCS4 and SOCS5 downregulation	[133]

**Table 4:** Natural products as anti-tumor compounds in GC therapy via targeting STAT3.

Anti-Tumor Compound	Cell Line	Dose	Duration of Experiment	Results	Refs
Piperine	TMK-1 human GC cell line	25, 50 and 100 μM	1 h	Downregulation of STAT3 Inhibition of IL-1β and IL-6 Decreasing viability and proliferation of cancer cells	[175]
Tanshinone IIA	Human GC cell lines (SNU-638, MKN1 and AGS)	2.5, 5 and 10 μg/mL	12, 24, 48 and 72 h	Inhibition of STAT3 Reduction in the progression and malignancy of cancer cells	[176]
Oxymatrine	Human GC cell lines SGC-7901, MGC-803, BGC-823, HGC-27, AGS and GES-1	0.5, 1, 2, 4 and 8 mg/mL	24, 48 and 72 h	Diminishing proliferation and malignancy of cancer cells Inhibition of IL-21R-mediated STAT3	[177]
Luteolin	Gastric tumor cell lines of SGC7901, SGC7901/DDP, HGC27, MGC803, BGC803 and BGC823	10 μM	-	Selective eradication of STAT3 overexpression-GC cells Increasing the binding of STAT3 to SHP-1	[178]
Parthenolide	Human GC drug-resistant SGC-7901/DDP cell line	1.25, 2.5, 5 and 10 μmol/L	24, 48 and 72 h	Induction of apoptosis Inhibition of drug resistance via the downregulation of STAT3	[179]
Curcumin analogue	Human GC cell lines (BGC-823, SGC-7901)	0.5, 1, 5, 10, 20, 50, 80 and 100 μM	24 and 48 h	Induction of apoptosis and mitotic arrest Downregulation of STAT3	[180]
Nifuratel	Human GC cell lines SGC-7901 and BGC-823	75, 150 and 300 μM	24 h	Inhibition of IL-6-mediated STAT3 activation	[181]
Cryptotanshinone	Human GC cell lines SGC-7901 and HGC-27	2.5, 5, 7.5, 10, 15 and 20 μM	4 h	Enhancing anti-tumor activity of doxorubicin Inhibition of STAT3 phosphorylation	[182]
Asiatic acid	SGC7901 (metastatic carcinoma of lymph node)	1, 5, 10, 25 and 50 μM	12 h	Stimulation of apoptosis Inhibition of proliferation and migration Downregulation of STAT3	[183]
Sulforaphane	Human GC cell lines MGC803 and BGC823	2.5, 5 and 10 μM	72 h	Sensitizing cancer cells to chemotherapy Suppressing cancer stem cell-like properties Upregulation of miR-124 and subsequent downregulation of IL-6/STAT3	[184]
Thymoquinone	Three human GC cells (HGC27, BGC823 and SGC7901)	25, 50 and 75 μmol/L	24 h	Suppressing STAT3 phosphorylation Downregulation of survival factors such as Bcl-2 and cyclin-D	[185]
Paeoniflorin	Human gastric carcinoma MGC-803 cells and human normal gastric mucosa GES-1 cell lines	5, 10 and 20 μmol/L	48 h	Downregulation of STAT3 Interfering with the proliferation and invasion of cancer cells	[186]
Eupatilin	Human GC cell line MKN45	50 and 100 μM	16 h	Inhibiting the STAT3 signaling pathway Suppressing VEGF and the growth of cancer cells	[187]
Epigallocatechin-6-gallate	Human gastric cancer (AGS) cells	5, 10, 25 and 50 μmol/L	24 h	Suppressing IL-6/STAT3/VEGF results in growth inhibition	[188]
Cucurbitacin B	GC MKN-45 cells	0.1, 1 and 10 μM	12, 24 and 48 h	Sensitizing cancer cells to cell death Downregulation of JAK2/STAT3	[189]
Ponicidin	Human MKN28 cell line	10, 25 and 50 μmol/L	48 h	Induction of apoptosis Downregulation of JAK2/STAT3	[190]
Cycloastragenol	Human gastric adenocarcinoma SNU-1 and SNU-16 cells	1, 5, 10, 30 and 50 μM	24 h	Inhibition of STAT3 phosphorylation at tyrosine 705 via suppressing Src and JAK1/2 activation Induction of apoptosis	[191]
Fucoxanthin	SGC-7901 cells	25, 50 and 75 μM	24 h	Downregulation of STAT3 Induction of apoptosis and cell cycle arrest	[192]
HJC0152 (niclosamide)	Six GC cell lines (AGS, HGC-27, MKN28, MKN45, SGC7901 and BGC-823)	5, 10 and 20 μM	1, 2 and 4 h	Suppressing the STAT3 signaling pathway and subsequent decrease in the expression of survival factors such as Survivin and Mcl-1	[193]
Piceatannol	Human GC SGC-7901 cell line	10 and 20 μM	-	Inhibiting STAT3 phosphorylation	[194]
BP-1-102	Five human GC cell lines (AGS, HGC-27, MKN28, MGC803 and SGC7901)	2, 4 and 6 μM	72 h	Suppressing the invasion and proliferation of cancer cells in a dose- and time-dependent manner Downregulation of STAT3	[195]

**Table 5:** lncRNAs as upstream modulators of STAT3 signaling pathway in GC cells.

lncRNA	Type of lncRNA	Downstream Signaling	Cell Line	Effect on STAT3	Major Results	Refs
SNHG16	Oncogene	MiR-135a/JAK2/STAT3	Four GC cell lines (BGC823, MGC803, MKN45, SGC7901) and normal GC cell line GES-1	Induction	Promoting colony formation and the proliferation of cancer cells Inhibition of apoptosis	[222]
HOTAIR	Oncogene	MiR-454-3p/STAT3/cyclin D1	AGS and SGC7901 cells	Induction	Knock-down of HOTAIR Sensitizing cancer cells to apoptosis Upregulation of miR-454-3p and the subsequent inhibition of the STAT3/cyclin D1 axis	[216]
PVT1	Oncogene	STAT3/VEGF	GES-1, SGC-7901, BGC-823, MNK-45, AGS, SUN-638, HGC-27 and HUVEC	Induction	Promoting angiogenesis and the growth of cancer cells	[219]
NEAT1	Oncogene	MiR-506/STAT3	BGC823, SGC-7901, AGS, MGC803, MKN28 cells, GES-1 and HEK-293T cells	Induction	Sponging miR-506 Enhancing expression of STAT3 Increasing the malignancy of cancer cells	[220]
GACAT3	Oncogene	IL-6/STAT3	Human GC cell lines HGC-27 and SGC-7901	Induction	Enhancing the proliferation of cancer cells in an inflammatory response behavior	[221]
HOXD-AS1	Oncogene	JAK2/STAT3	Human GC cell lines (SGC-7901, BGC-823, MGC803 and MKN-45)	Induction	Silencing of HOXD-AS1 is correlated with the downregulation of STAT3 and growth inhibition	[223]

**Table 6:** Molecular pathways targeting STAT3 in GC cells.

Signaling Network	Cell Line	Effect on STAT3	Results	Refs
CXCR4/JAK2/STAT3/VEGF	Human SGC-7901 and MKN45 cells	Induction	Induction of VEGF by CXCR4 Subsequent activation of JAK2/STAT3 Enhancing the migration and proliferation of cancer cells	[285]
NOX4/JAK2/STAT3/EMT	Six human GC cell lines (MKN-45, SGC-7901, MGC-803, BGC-823, MKN-28 and AGS)	Induction	Induction of JAK2/STAT3 by NOX4 Stimulation of EMT Promoting invasion	[286]
DC-SIGNR	Human GC cell lines, SGC-7901, MGC-803, BGC-823 and AGS, and the control gastric epithelial cell line GES-1	Induction	Ensuring the growth and viability of cancer cells by the induction of the JAK2/STAT3 pathway	[287]
HOXA11	GC cell lines (KATO III, NCI-N87, SNU-16, AGS and SNU-16) and HEK 293T	Induction	Promoting the stemness and migration of cancer cells by the stimulation of STAT3	[288]
TFF1/STAT3	AGS cells	Induction	Enhancing the proliferation and migration of cancer cells	[67]
DARPP-32/IGF-1R/STAT3	AGS cells	Induction	Promoting proliferation and invasion	[289]
BMX-ARHGAP/STAT3	Four human GC cell lines (SNU-5, MNK-45, AGS and SGC7901) and the normal gastric epithelial cell line (GES-1)	Induction	Maintaining the carcinogenesis ability of GC stem cells	[279]
CXCL16/STAT3/Ror1	MKN45, MKN45-Luc and KATOIII cells	Induction	Increasing progression and malignancy	[290]
Complement C3/JAK2/STAT3	Human SGC-7901 and MGC-803 cells, normal gastric epithelial cells (GES-1)	Induction	Poor prognosis and enhanced proliferation of cancer cells	[291]
BTF3/JAK2/STAT3/EMT	Human gastric epithelial cell line GES-1 and human GC cell lines, including AGS, HGC-27, MKN-28, MGC-803 and SGC-7901 cells	Induction	Induction of JAK2/STAT3 by BTF3 Stimulation of EMT Enhancing the proliferation and migration of cancer cells	[292]
IGF1/IGF1R/STAT3/IFITM2	GC cell lines	Induction	Enhancing the growth and metastasis of cancer cells	[293]
IL-6/JAK2/STAT3	GC cell lines SNU-1, MKN45, SGC7901 and MKN28	Induction	Secretion of IL-6 by CAMs Stimulation of JAK2/STAT3 by IL-6	[294]
TNF-α/IL-6/STAT3	SGC7901 cells	Induction	Induction of EMT Ensuring the metastasis of cancer cells	[295]
Succinate/STAT3/VEGF	Human gastric mucosal epithelial cell line GES-1 and human GC cell lines AGS (low-differentiated human gastric adenocarcinoma), NCI-N87 (well-differentiated human carcinoma), BGC-823 (low-differentiated human gastric adenocarcinoma) and SGC-7901 (moderate-differentiated human gastric adenocarcinoma)	Induction	Induction of VEGF by succinate via STAT3 overexpression Increasing viability and invasion of cancer cells	[296]
Cyclophilin B/STAT3/miR-520d-5p	GC cell lines	Induction	There is feedback consisting of the downregulation of miR-520d-5p and upregulation of cyclophilin B and STAT3, leading to the enhanced growth of cancer cells	[297]
CMTM3/STAT3/Twist1/EMT	Human GC cell line SGC-7901	Inhibition	Downregulation of STAT3 by CMTM3 Suppressing metastasis	[298]
HCCR/STAT3	Human GC cell lines AGS, MKN-45, BGC823, MGC803, HGC27, SGC7901, NCI-N87	Induction	Triggering chemoresistance	[299]
GRIM19/STAT3	Immortalized normal gastric epithelial cell line GES-1, human embryonic kidney HEK-293 cells, human GC SGC-7901 and BGC-823 cell lines	Inhibition	Suppressing STAT3 Induction of apoptosis	[300]
ROS/IL-6/STAT3	AGS cells	Induction	Enhanced generation of ROS by *H. pylori* Stimulation of IL-6/STAT3 Enhanced proliferation and invasion of cancer cells	[301]
IL-6/STAT3/VEGF	GC cell lines including SGC-7901, MGC, MKN-28 and AGS	Induction	Promoting invasion and angiogenesis Induction of STAT3 and subsequent activation of VEGF	[302]
IL-17/STAT3/VEGF	Human GC (AGS) cells and other cells SGC7901, MKN 45 and BGC823	Induction	Enhancing growth Induction of angiogenesis Activation of STAT3/VEGF	[303]

## Abbreviations

GC	gastric cancer
*H. pylori*	*Helicobacter pylori*
EBV	Epstein–Barr virus
lncRNAs	long non-coding RNAs
miR	microRNA
EMT	epithelial-to-mesenchymal transition
STAT	signal transducer and activator of transcription
SH_2_	Src homology-2
TAD	transcription activation domain
JAKs	Janus kinases
SOCS	suppressor of cytokine signaling
PIAS	protein inhibitor of activated STAT
SLP-2	stomatin-like protein 2
CTM	Chinese traditional medicine
VEGF	vascular endothelial growth factor
MMPs	matrix metalloproteinases
ECM	extracellular matrix
ROS	reactive oxygen species
ER	endoplasmic reticulum
APG	apigetrin
TXN	troxerutin
DHA	docosahexaenoid acid
PPAR-γ	peroxisome proliferator-activated receptor gamma
SIRT	sirtuin
STMN	stathmin
S1PR1	sphingosine-1-phosphate receptor
YB-1	Y-box binding protein-1
NFIB	nuclear factor I/B
TME	tumor microenvironment
CAMs	cancer-associated macrophages
TAMs	tumor-associated macrophages
CAFs	cancer-associated fibroblasts
MSK1	mitogen- and stress-activated protein kinase 1
EZH2	enhancer of zeste homolog 2
